# Physiological and transcriptional response to drought stress among bioenergy grass *Miscanthus* species

**DOI:** 10.1186/s13068-021-01915-z

**Published:** 2021-03-06

**Authors:** Jose J. De Vega, Abel Teshome, Manfred Klaas, Jim Grant, John Finnan, Susanne Barth

**Affiliations:** 1 Earlham Institute, Norwich Research Park, Norwich, NR4 7UZ UK; 2 John Innes Centre, Norwich Research Park, Norwich, NR4 7UH UK; 3 Teagasc Crop Science Department, Oak Park, Carlow, R93XE12 Ireland; 4 Feed and Forage Development, International Livestock Research Institute (ILRI), Addis Ababa, Ethiopia; 5 Teagasc Statistics and Applied Physics Research Operations Group, Ashtown, Dublin 15, D15 DY05 Ireland

**Keywords:** Differentially expressed genes (DEGs), Drought, Electrolyte leakage, Gene ontology, Miscanthus, Relative water content, RNA-seq

## Abstract

**Background:**

*Miscanthus* is a commercial lignocellulosic biomass crop owing to its high biomass productivity, resilience and photosynthetic capacity at low temperature. These qualities make *Miscanthus* a particularly good candidate for temperate marginal land, where yields can be limited by insufficient or excessive water supply. Differences in response to water stress have been observed among *Miscanthus* species, which correlated to origin. In this study, we compared the physiological and molecular responses among *Miscanthus* species under excessive (flooded) and insufficient (drought) water supply in glasshouse conditions.

**Results:**

A significant biomass loss was observed under drought conditions in all genotypes. *M. x giganteus* showed a lower reduction in biomass yield under drought conditions compared to the control than the other species. Under flooded conditions, biomass yield was as good as or better than control conditions in all species. 4389 of the 67,789 genes (6.4%) in the reference genome were differentially expressed during drought among four *Miscanthus* genotypes from different species. We observed the same biological processes were regulated across *Miscanthus* species during drought stress despite the DEGs being not similar. Upregulated differentially expressed genes were significantly involved in sucrose and starch metabolism, redox, and water and glycerol homeostasis and channel activity. Multiple copies of the starch metabolic enzymes BAM and waxy GBSS-I were strongly up-regulated in drought stress in all *Miscanthus* genotypes, and 12 aquaporins (PIP1, PIP2 and NIP2) were also up-regulated in drought stress across genotypes.

**Conclusions:**

Different phenotypic responses were observed during drought stress among *Miscanthus* genotypes from different species, supporting differences in genetic adaption. The low number of DEGs and higher biomass yield in flooded conditions supported *Miscanthus* use in flooded land. The molecular processes regulated during drought were shared among *Miscanthus* species and consistent with functional categories known to be critical during drought stress in model organisms. However, differences in the regulated genes, likely associated with ploidy and heterosis, highlighted the value of exploring its diversity for breeding.

**Supplementary Information:**

The online version contains supplementary material available at 10.1186/s13068-021-01915-z.

## Background

The global challenge of feeding the ever-increasing world population is exacerbated when food crops are being used as feedstock for green energy production [1]. Therefore, plant species for ethanol and chemical production should prioritise the following attributes; being nonfood related, perennial, and able to grow on marginal lands, having high biomass yield, low chemical and mechanical input requirement, and enhanced water-use efficiency and high carbon storage capacity [2–4]. Amongst grass species, *Miscanthus* species fulfil most of the qualities above.

*Miscanthus* spp. are semi-domesticated rhizomatous perennial C_4_ grass species, originally from Eastern Asia [1]. *Miscanthus* species have been used as forage species in Japan, Korea and China for thousands of years [5, 6]. Because of its high biomass yield and high ligno-cellulose content, *Miscanthus* spp. are presently commercially used as feedstock for bioenergy production [7–10]. The sterile triploid *M. x giganteus* hybrid (3*n* = 57, *x* = 19), *M. sacchariflorus* (2*n* = 4*x* = 76) and *M. sinensis* (2*n* = 2*x* = 38) highly performing accessions, and newly bred hybrids between *M. sacchariflorus* and *M. sinensis* are commercially grown as biomass feedstock [11–13]. A decade-long trial in Europe showed that *Miscanthus x giganteus* produced up to 40 tonnes of dry matter per hectare and year after 2 years of establishment [14]. A study on its biofuel capacity showed that *Miscanthus* was more efficient in ethanol production per hectare than switchgrass and corn [15].

*Miscanthus* species are an ideal biofuel crop in temperate marginal land because of an outstanding resilience and photosynthetic capacity at low temperature ca. 5 °C [16, 17]. However, yields may be limited by insufficient or excessive water supply, and plant survival is endangered under extreme summer drought [18]. Differences in osmotic adaptation to water stress were observed among *Miscanthus* ecotypes, which correlated with the annual rainfall and microclimate at each genotype’s original location [19, 20].

Differences in physiological response to drought stress were observed among *Miscanthus* species. In a glasshouse study on *M. x giganteus* where water supply was restricted, a reduction in stem elongation rate was the primary response [21]. Furthermore, a reduction in photosynthetic performance (chlorophyll content of leaves) and plant water status (leaf relative water content) were also observed during the same experiment. In a pot study under reduced water supply conditions, *M. sacchariflorus* had the highest dry matter per plant, followed by *M. x giganteus* [18]. On the contrary, little is known about the productivity of *Miscanthus* under flooded and moisture-saturated soil conditions commonly experienced on marginal lands.

Previous differential expression studies carried out in *Miscanthus* species have allowed the identification of transcripts and molecular mechanisms under different water stress conditions [22–24]. Previous studies, however, did not compare the response among different *Miscanthus* species. A RNA-seq analysis with one drought-tolerant accession of *M. sinensis* in a time series with six collection time points between zero and 60 days of drought stress revealed that a 15-day period is a threshold to trigger a cascade of responses under water deficit stress [22]. Five accessions of *M. lutarioriparius* were collected in China and exposed to salt-induced osmotic stress. A RNA-seq analysis identified population-specific and shared response genes associated with photosynthesis, osmosis adjustment and signal transduction during osmotic stress [23, 24].

In this study, we compared the physiological responses among three *Miscanthus* species and a newly bred interspecific triploid hybrid in water flooded and drought conditions. The induced physiological conditions were used for an in-depth transcriptome study on the molecular basis of water stress in *Miscanthus* species. Our results will contribute to understand differences in tolerance among these species and facilitate future genomics assisted breeding in *Miscanthus*.

## Results

### Phenotypic differences during water stress

**Fig. 1:**
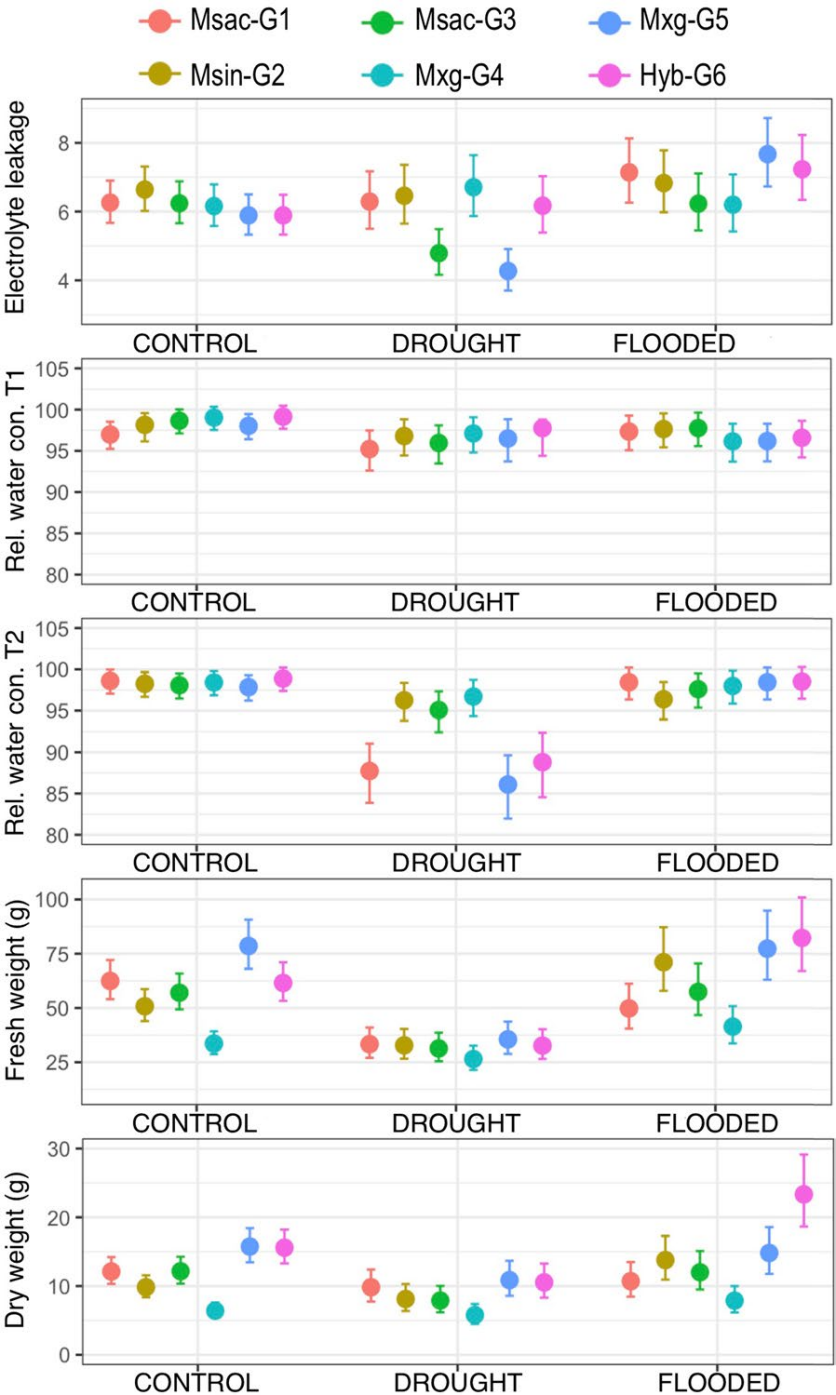
Distribution of phenotypic measurements for electrolyte leakage (logarithmic values), relative water content (logarithmic values) at two time points, and fresh and dry biomass weight [in grammes, (logarithmic values)] for each genotype across the control, drought and flooded conditions

Two *M. sacchariflorus* (Msac-G1 and Msac-G3), one *M. sinensis* (Msin-G2), two *M. x giganteus* (Mxg-G4 and Mxg-G5) and a newly bred interspecific triploid hybrid (Hyb-G6) were compared in drought and flooded conditions. Responses of each genotype were evaluated in terms of electrolyte leakage, relative water content (RWC) at two time points, and fresh and dry biomass weight (Fig. 1; Additional file 1: Figure S1: fresh and dry biomass by genotype with non-log transformed data).

#### Electrolyte leakage

Significant effects (*P* < 0.05) on electrolyte leakage (logarithmic value) were observed for all contrasts in the experiment (Table 1). Genotype × treatment interaction effects were significant at *P* < 0.001. When comparing drought, flooded and control conditions, high mean electrolyte leakage was recorded for Msac-G1, Msin-G2, Mxg-G5 and Hyb-G6 under flooded conditions, indicating stress induced by excess water. On the contrary, lower mean electrolyte leakage was recorded for Msac-G3 and Mxg-G5 in both control and drought conditions (Fig. 1, Table 2, Additional file 2: Table S1).

**Table 1:** Analysis of variance (REML method), fixed effects are displayed for electrolyte leakage, relative water content, fresh weight and dry weight

Effects	Electrolyte leakage	Relative water content	Fresh weight	Dry weight
Treatment	0.0204	0.0039	< 0.0001***	0.0012**
Genotype	0.0061	0.0039	< 0.0001***	< 0.0001***
Genotype × treatment	0.0002	0.0005	0.0163*	0.0862 ns
Date	NA	0.0165	NA	NA
Date × genotype	NA	0.5691	NA	NA
Treatment × genotype × date	NA	0.0111	NA	NA

*NA not applicable; **p > 0.01; ***p > 0.001

**Table 2:** Estimates and confidence intervals (brackets) of electrolyte leakage, relative water content, fresh biomass, dry biomass and fresh biomass for six genotypes and three conditions (control, drought, flooded)

Genotype	Condition	Electrolyte leakage (25/09/13)	Relative water content T1 (13/09/13)	Relative water content T2 (24/09/13)	Fresh weight-g-(25/09/13)	Dry weight-g-(27/09/13)	RNA-seq libraries
(G1) *M. sacchariflorus* var. Dk-1	Control (C)	6.26 (5.67, 6.90)	96.98 (95.24, 98.53)	98.60 (97.06, 99.97)	62.44 (54.06, 72.08)	12.12 (10.32, 14.21)	M39, M51, M63
	Drought (D)	6.29 (5.50, 7.17)	95.22 (92.61, 97.47)	87.73 (83.87, 91.04)	33.33 (27.05, 41.00)	9.83 (7.75, 12.40)	M35, M47, M59
	Flooded (W)	7.14 (6.26, 8.13)	97.34 (95.08, 99.28)	98.43 (96.35, 100.22)	49.81 (40.53, 61.17)	10.71 (8.46, 13.49)	M31, M43, M55
(G3) *M. sacchariflorus*	Control (C)	6.24 (5.66, 6.88)	98.65 (97.12, 100.02)	98.07 (96.46, 99.49)	57.02 (49.36, 65.84)	12.16 (10.35, 14.25)	NA
	Drought (D)	4.79 (4.16, 5.49)	95.96 (93.47, 98.10)	95.07 (92.42, 97.33)	31.38 (25.46, 38.62)	7.90 (6.19, 10.02)	NA
	Flooded (W)	6.23 (5.45, 7.11)	97.76 (95.57, 99.64)	97.59 (95.37, 99.50)	57.43 (46.75, 70.49)	12.00 (9.50, 15.09)	NA
(G2) *M. sinensis* var. genotype-48	Control (C)	6.64 (6.02, 7.31)	98.15 (96.15, 99.57)	98.25 (96.67, 99.66)	50.80 (43.96, 58.67)	9.84 (8.36, 11.57)	M40, M52, M64
	Drought (D)	6.46 (5.65, 7.36)	96.80 (94.44, 98.82)	96.25 (93.81, 98.35)	32.80 (26.62, 40.36)	8.12 (6.37, 10.29)	M36, M60
	Flooded (W)	6.83 (5.98, 7.78)	97.64 (95.43, 99.54)	96.37 (93.95, 98.45)	71.09 (57.92, 87.21)	13.78 (10.94, 17.29)	M32, M44, M56
(G4) *M. x giganteus*	Control (C)	6.16 (5.58, 6.79)	99.03 (97.54, 100.35)	98.41 (96.85, 99.80)	33.60 (28.72, 39.27)	6.41 (5.40, 7.59)	NA
	Drought (D)	6.71 (5.87, 7.64)	97.11 (94.81, 99.08)	96.70 (94.33, 98.73)	26.48 (21.46, 32.62)	5.79 (4.48, 7.40)	NA
	Flooded (W)	6.20 (5.42, 7.08)	96.15 (93.69, 98.29)	97.99 (95.84, 99.84)	41.41 (33.66, 50.89)	7.88 (6.17, 10.00)	NA
(G5) *M. x giganteus* cv. Illinois	Control (C)	5.89 (5.33, 6.50)	98.03 (96.42, 99.46)	97.84 (96.21, 99.29)	78.57 (68.07, 90.67)	15.75 (13.45, 18.42)	M41, M53, M65
	Drought (D)	4.27 (3.70, 4.91)	96.50 (93.72, 98.83)	86.09 (81.96, 89.63)	35.54 (28.86, 43.71)	10.86 (8.58, 13.68)	M37, M49, M61
	Flooded (W)	7.67 (6.73, 8.72)	96.19 (93.73, 98.29)	98.43 (96.35, 100.22)	77.32 (63.01, 94.83)	14.81 (11.77, 18.57)	M33, M45, M57
(G6) *Miscanthus* interspecific triploid hybrid (‘3n’)	Control (C)	5.89 (5.33, 6.49)	99.15 (97.68, 100.46)	98.88 (97.38, 100.22)	61.52 (53.27, 71.02)	15.58 (13.30, 18.21)	M42, M54, M66
	Drought (D)	6.17 (5.39, 7.03)	97.76 (94.40, 98.79)	88.80 (84.55, 92.37)	32.68 (26.51, 40.21)	10.53 (8.31, 13.27)	M38, M50, M62
	Flooded (W)	7.23 (6.34, 8.23)	96.59 (94.20, 98.64)	98.51 (96.44, 100.29)	82.27 (67.05, 100.89)	23.34 (18.66, 29.13)	M34, M46, M58

#### Relative water content (RWC)

Significant effects (*P* < 0.05) on RWC (logarithmic value), were observed for all contrasts in the experiment except for block effects and date x genotype treatment interaction (Table 1). RWC was recorded at two time points and significant reduction in RWC was observed at the second time point (Fig. 1). No significant difference in RWC was observed between control and flooded conditions at the second time point.

#### Fresh biomass weight

Highly significant effects (*P* < 0.05) on fresh biomass weight (logarithmic value), were observed for all contrasts in the experiment (Table 1). Mean fresh biomass was higher in flooded conditions for genotypes Msin-G2, Msac-G3, Mxg-G4 and Hyb-G6 (Fig. 1 and Table 2). Mxg-G5 had the highest mean fresh weight both in control and drought conditions, and the second highest in flooded conditions after Hyb-G6.

#### Dry biomass weight

Highly significant effects (*P* < 0.01) for dry biomass weight (logarithmic value) were observed for all contrasts in the experiment (Table 1). As was observed for fresh biomass weight, dry biomass was significantly reduced under drought conditions. Mxg-G5 had the highest mean dry biomass both in control and drought conditions (Fig. 1 and Table 2).

### RNA-seq analysis under water stress

Four genotypes (Msac-G1, Msin-G2, Mxg-G5 and Hyb-G6) were selected because of sequencing budget constraints by discarding one of the two genotypes from the same species. Genotypes were sampled towards the end of the experiment and sequenced in 2014. The number of raw reads from each library ranged from 16.9 to 42.4 M, and a total of 945.2 M reads were obtained (Additional file 3: Table S2). After filtering out adaptor sequences and ambiguous and low-quality reads, clean reads totalled 926.8 M for all samples. Alignment and mapping summary for each library is presented in Additional file 3: Table S2 and read-counts per gene in Additional file 4: Table S3.

**Fig. 2:**
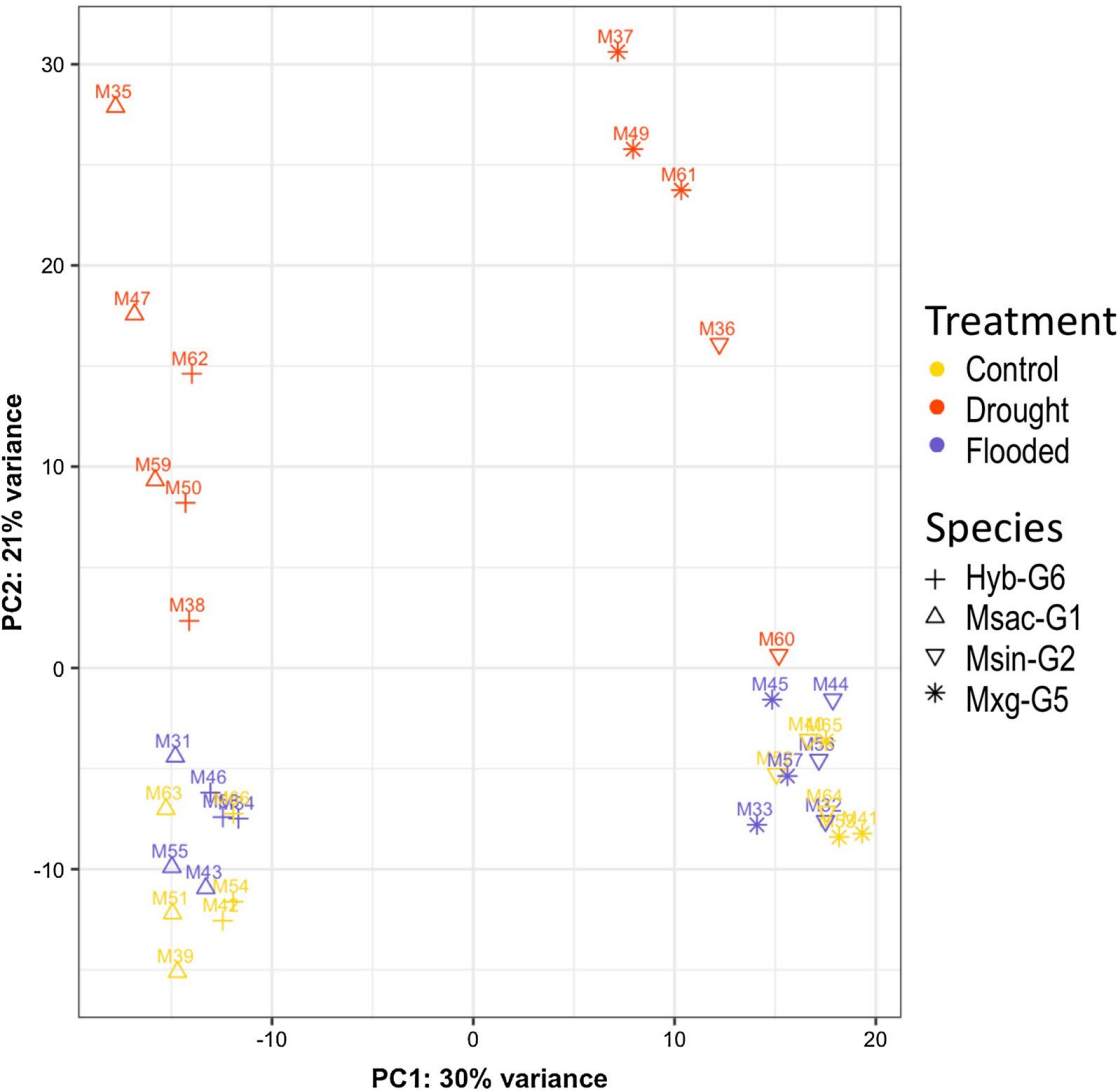
Principal component analysis of the normalised gene counts from RNA-seq libraries generated from four *Miscanthus* species in control, drought and flooded conditions

When the normalised counts (Additional file 5: Table S4) were used to cluster the samples (Fig. 2), the samples clustered firstly by species (PC1: 30% variance) and later by condition (PC2: 21% variance). Msac-G1 and the Hyb-G6 clustered together and separated from Msin-G2 and Mxg-G5, which clustered together. However, differences resulting from the treatment were only observed for drought samples. Control and flooded samples clustered together and away from drought samples, except for one drought sample (M48) from Msin-G2*,* which was discarded from down-stream analysis (Fig. 2).

### Effects of drought on *Miscanthus* transcriptomes

**Fig. 3:**
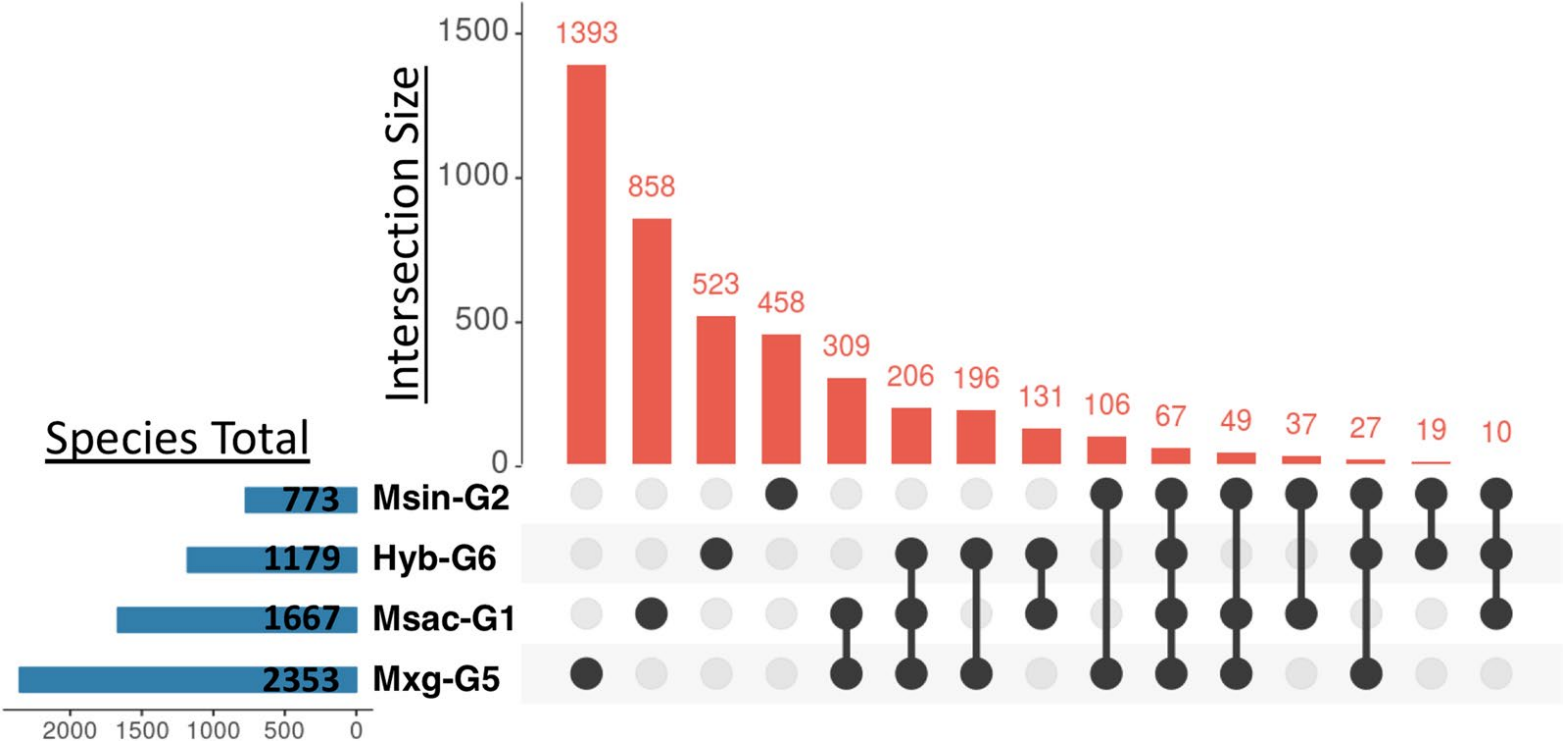
Number of differentially expressed genes (DEGs) shared (connected by black dots) within and among four *Miscanthus* species under drought conditions

A total of 4389 of the 67,789 genes (6.4%) in the reference genome were significantly differentially expressed in total (Fig. 3 and Additional file 6: Table S5). The highest number of DEGs was observed in Mxg-G5 (2353) and the lowest in Msin-G2 (773). The UpSet diagram highlights shared DEGs among the four Miscanthus species under drought situation (Fig. 3). Only 67 DEGs were shared by all four genotypes. On the contrary, 3,232 of the 4389 DEGs (73.3%) were differentially expressed in a single genotype. On the other hand, only 134 differentially expressed genes were detected in flooded against control conditions and none of those were shared among all genotypes (Additional file 7: Figure S2 and Additional file 8: Table S6).

### Enriched gene ontology (GO) terms in DEGs during drought

**Fig. 4:**
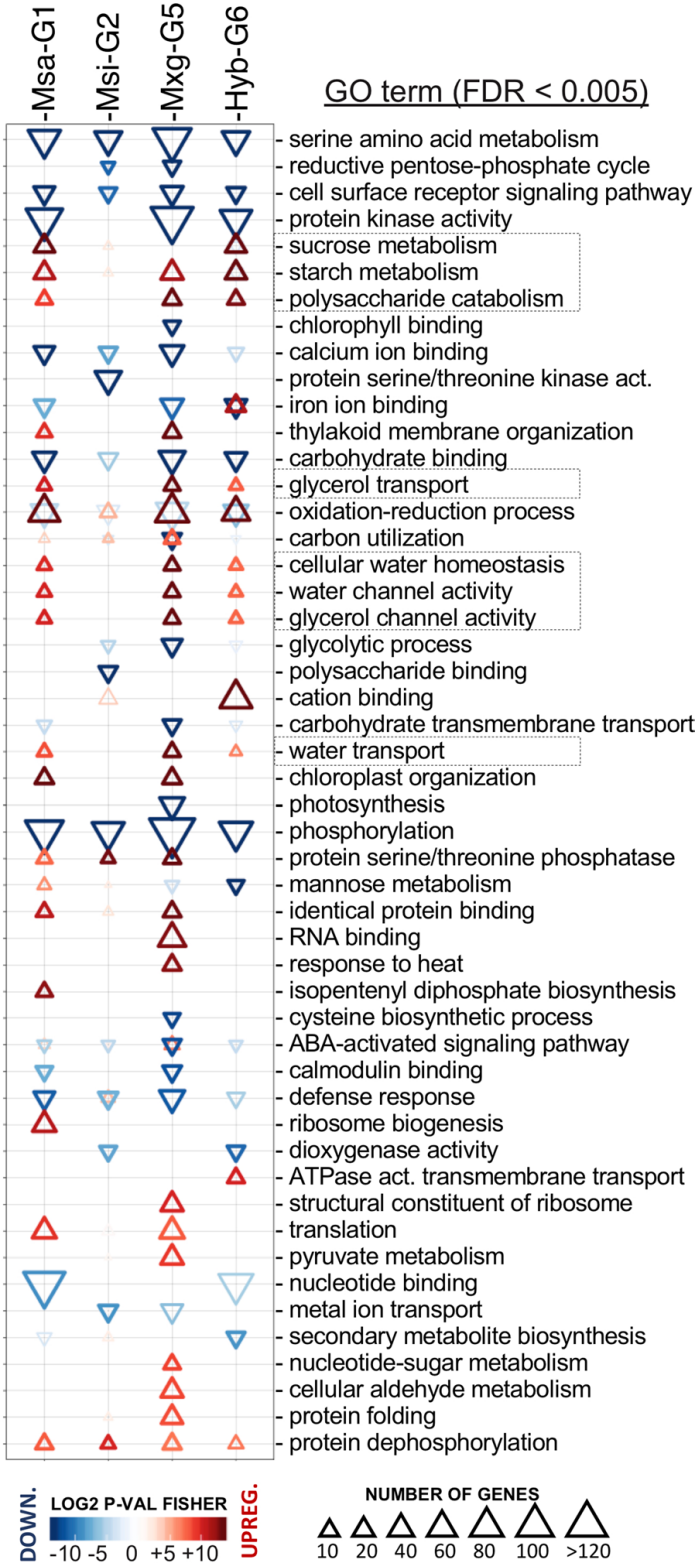
GO terms (rows) that were significantly enriched (*P* < 0.005) in each *Miscanthus* species (columns) among either up-regulated (top-pointing triangles) or down-regulated (bottom-pointing triangles) differentially expressed genes (DEGs) in drought conditions. The size of a triangle is proportional to the number of DEGs annotated with that GO term. Rows are sorted by descending *P*-value (F-fisher test) and the triangle colour is representative to the obtained *P*-value, from lower (dark colour) to higher (light colour). Yellow (*P* > 0.05) and white (*P* > 0.1) triangles were not significantly enriched

Enrichment analysis of GO terms over-represented among DE genes allowed us to identify the biological processes (BP) and molecular functions (MF) that are regulated in each genotype during drought. Firstly, we annotated the reference transcriptome with GO and GO-SLIM terms (Additional file 9: Table S7). The same biological processes were regulated in all the genotypes in the same direction (either up- or down-regulated) and by a similar-enough number of DEGs (Additional file 10: Tables S8 and Additional file 11: Table S9). This is also evidenced by the similar shape sizes (number of genes), colours (red for up-regulation and blue for down-regulation) and intensities (darker for lower *p*-values) in Fig. 4.

Downregulated differentially expressed genes were significantly enriched in GO terms involved in protein phosphorylation and kinase activity, cell receptor signalling, amino acid metabolism, and ion binding; while up-regulated differentially expressed genes were significantly enriched in GO terms involved in sucrose and starch metabolism, redox, and water and glycerol homeostasis and channel activity (Fig. 4). DE genes in these functional categories were functional annotated (Additional file 12: Table S10). Relevant functions (water homeostasis and channelling, and starch and sucrose synthesis) were further characterised in the next result sections. A similar analysis of the GO slim terms among DEGs enriched during drought highlighted a wider spread of GO terms in functions similar to the ones analysed previously (Additional file 13: Figure S3).

### Candidate genes involved in starch and sucrose synthesis and degradation

We observed a cluster of three related GO terms (“sucrose metabolism”, “starch metabolism” and “polysaccharide catabolism”), which was up-regulated with strong *p*-values during drought stress and contained a similar number of genes among genotypes (dotted box in Fig. 4). However, sucrose metabolism was not enriched in Mxg-G5 and none of these GO terms was enriched in Msin-G2 (most enriched GO terms were not enriched in Msin-G2, Fig. 4). The cluster of related GO terms included 53 DEG in total (Additional file 14: Table S11). Thirty-five of these genes could be mapped to reactions in the starch and sucrose KEGG pathways (Additional file 15: Figure S4).

Twelve genes were involved in the direct degradation of starch to maltose (Enzymatic codes -EC- 3.2.1.1, 3.2.1.2, and 3.2.1.68). Ten genes were homologous to BAM1 (Additional file 14: Table S11; EC 3.2.1.2) and highly up-regulated with 3.4- to 8.9-fold change expressions (FC), six of them were common among the genotypes. Involved in the same process, AMY3 (T282800; EC 3.2.1.1) had a low 1 to 1.3-fold-change expression (FC), and ISA3 (4G215400; EC 3.2.1.68) was weakly up-regulated among the genotypes (0.2–1 FC). On the other hand, two DE glycogen phosphorylase genes were involved in the first step of the degradation of starch in glucose (2.4.1.1), but only one (1G063200) was up-regulated in Mxg-G5 (3.1 FC) and less so in the new interspecific triploid hybrid (1.6 FC) and Msac-G1 (0.7 FC). The related SEX1 gene (18G152900; EC 2.7.9.4) showed a very similar expression pattern; more strongly up-regulated in Mxg-G5 (2.4 FC) than in Hyb-G6 (1.3 FC) or Msac-G1 (0.8 FC).

Concerning starch biosynthesis, waxy gene GBSS-I (19G002300), which synthetises amylose -a starch precursor-, showed very high up-regulation in Msac-G1 (11.9 FC), Mxg-G5 (8.8 FC) and Hyb-G6 (9.3 FC). Two genes involved in the ADP-glucose to starch synthesis, SS3 (T393000; EC 2.4.1.21) and BE1 (5G197100; EC 2.4.1.21) were moderately up-regulated in these three genotypes (1.4–2.3 FC and 0.51–1.8 FC, respectively). SUS3 (1G358800; EC 2.4.1.13) and two genes encoding SPS1F (16G229500 and 17G242300; 2.4.1.14), which are involved in the last steps of sucrose synthesis, were up-regulated in all genotypes. SUS3 fold-change expression was 1.8–2.6 FC, while SPS1F was 0.52–1.3 FC.

Five cellulose synthase genes involved in secondary cell wall biosynthesis (CESA4 and IRX1/3, Additional file 14: Table S11; EC 2.4.1.12) were strongly up-regulated in Mxg-G5 (5.2–10.4 FC), two were also strongly up-regulated in Mxg-G5 and Hyb-G6 (4.7 and 9.2 FC), but none was in the other genotypes. One glycosyl hydrolase 9B5 (GH9B5) involved in cellulose degradation (3G236400; EC 3.2.1.4) was up-regulated in Msac-G1 and Hyb-G6 (2 and 1.7 FC, respectively), but highly up-regulated in Mxg-G6 (3.7 FC).

### Candidate genes involved in water homeostasis and channelling

We observed a cluster of five related GO terms (“cellular water homeostasis”, “water channel activity”, “water transport”, “glycerol transport”, and “glycerol channel activity”), which was up-regulated with strong *P*-values during drought stress and contained a similar number of genes among genotypes (dotted box in Fig. 4). Within these GO terms in any genotype, there were thirteen genes in total, twelve of them were aquaporins, and one (18G085200) was homologous to the LRR kinase EREC1/TE1 (“Transpiration efficiency 1”; Additional file 16: Table S12).

Using rice as a reference, three aquaporins were homologous to PIP2-1 (3G107200), PIP2-2 (7G413400), and PIP2-7 (4G263800). PIP2-7 was lowly up-regulated in all genotypes (0.2–1 FC), while PIP2-2 was only up-regulated in Mxg-G6 (3.6 FC), and PIP2-1 was up-regulated in Msac-G1 too (1.2–1.6 FC). Four aquaporins were homologous to PIP1; The homologous genes to PIP1-1 (7G437200) and PIP1-3 (7G548500) were clear, and two additional genes (8G232800 and 12G174400) were homologous to other PIP1 proteins. PIP1-3 (7G548500) was strongly up-regulated in Msac-G1 (5.3 FC), Mxg-G5 (3.7 FC) and Hyb-G6 (7.3 FC). NIP2 (7G481100) was highly up-regulated in all genotypes (1.84–2.7 FC) and LRR kinase ER1 (EC 2.7.11.1; ERECTA homolog 1) was only DE in Mxg-G5 with a low up-regulation (0.2 FC). Four aquaporins had no characterised homologous genes: 1G219200, 3G326300, 8G270100, T569700. The uncharacterised aquaporin 3G326300 was strongly up-regulated in Msac-G1 (7.7 FC) alone, but was wholly absent in the triploid hybrid. The uncharacterised aquaporin 1G219200 was only up-regulated in Mxg-G5 (4.7 FC). All thirteen genes were highly up-regulated in Mxg-G5, but only half of them were in Msac-G1 and Hyb-G6 (Additional file 14: Table S11).

## Discussion

Physiological differences in osmotic adaptation to water stress were observed among *Miscanthus* ecotypes, which correlated with the annual rainfall and microclimate at each genotype’s original location [19, 20]. The present study focused on comparing the physiological and transcriptional responses in six genotypes from different *Miscanthus* species, *M. sacchariflorus*, *M. sinensis,* their natural hybrid *M. x giganteus*, and a new interspecific triploid hybrid, when subjected to water deficit (drought) and waterlogging (flooded).

### Comparative physiological response to water stress in *Miscanthus*

Electrolyte leakage is an indicator of plant tissue damage when plant cells are exposed to abiotic stresses, such as drought [25]. In the present study, ANOVA for electrolyte leakage (logarithmic value) revealed a significant difference among the six genotypes in the three conditions (Table 1). Reduced electrolyte leakage under stress conditions is positively associated with the plant’s capacity to tolerate the stress in the given time [26]. Under drought conditions, the lower mean electrolyte leakage was recorded for Mxg-G5 (Table 2).

Several studies, both in greenhouse and field conditions, reported that water deficit reduces photosynthetic capacity and hence a significant yield loss in *Miscanthus* [17, 20, 21, 27]. In the present study, biomass yield (fresh or dry weight) was significantly reduced for all genotypes under drought conditions, in line with previous findings (Table 2; Additional file 1: Figure S1). The highest biomass yield (fresh and dry weight) was recorded for Mxg-G5 in control and drought conditions. A similar result was reported earlier [18], where a *M. x giganteus* genotype had the second highest dry matter per plant in a pot study with reduced water supply. Remarkably, the newly bred interspecific triploid hybrid (Hyb-G6) had the highest mean fresh and dry weight under flooded conditions, suggesting its capacity to thrive under waterlogging conditions. *M. lutarioriparius* was the highest yielding among different *Miscanthus* species evaluated across different agro-ecological region in China [28]. However, a genotype from this species was not included in our study.

We also measured relative water content (RWC) at two time-points (Fig. 1; Table 2). A 5% reduction in RWC can lead to a 40-to-50% reduction in photosynthesis [29]. ANOVA among genotypes revealed a significant difference in relative water content (RWC) between genotypes and treatment groups (*P* < 0.05; Table 1). The highest mean RWC at both time-points in drought was recorded for Mxg-G4 (Table 2). A 10% reduction in mean RWC content in Mxg-G5 did not appear to significantly affect biomass, Mxg-G5 had the highest mean fresh and dry weight in drought conditions, as previously discussed. Our results in *M. x giganteus* under drought conditions contradicted previous results where *M. x giganteus* showed a lower water-use efficiency than its progenitors, *M. sinensis* and *M. sacchariflorus* [17, 18, 20]. However, such disparity could arise from differences in genetic diversity among genotypes of the same species.

### Changes in transcript expression under water stress in *Miscanthus*

Four samples from four *Miscanthus* species were evaluated by comparing transcriptome changes under control, drought and flooded conditions. From a total of 67,789 transcripts, 4,389 (6.4%) were differentially expressed in drought conditions (Fig. 3). The highest number of DEGs were observed for a *M. x giganteus* genotype (Mxg-G5; 2353 genes), which also showed a lower reduction in biomass yield than the other genotype under drought conditions compared to the control. We obtained almost half of the DEG in *M. sinensis* (Msin-G2) than in *M. sacchariflorus* (Msac-G1). In our study, we only analysed leaf tissues. However, a transcriptomics study in water-deficit conditions in sorghum showed that the number of DEGs in root samples is much larger than those observed in leaf samples [30].

All four genotypes showed no significant differences in their transcriptome profile when exposed to flooded conditions (Additional file 7: Figure S2). PCA also revealed a similar result, since no clear separation was observed between control and flooded samples (Fig. 2). The phenotypic assay corroborates this result; both fresh and dry weight measurements were equal or higher in flooded conditions compared to the control for most genotypes (Fig. 1). Although the present study was conducted under glasshouse conditions, the positive performance of *Miscanthus* genotypes in flooded conditions indicates that *Miscanthus* could perform well in saturated fields.

### Functional categories associated with drought conditions in *Miscanthus*

Gene ontology (GO) enrichment analysis allowed us to explore the functions related to drought-responsive genes in *Miscanthus*. While most of the genes were differentially expressed in a single genotype (Fig. 3), the enrichment analysis of GO terms revealed that the same biological processes were regulated in all the genotypes during stress conditions (Fig. 4). No enriched functional category was observed for the *M. x giganteus* (Mxg-G5) and the triploid hybrid (Hyb-G6) genotypes (both sharing ploidy and parental species) that was not also seen in the *M. sacchariflorus* genotype (Msac-G1). Most functional categories were not enriched in the *M. sinensis* genotype (Msin-G2), where we did observe less DEGs in the first place.

Sucrose and starch synthesis and degradation were up-regulated with strong *p*-values during drought stress in all genotypes. The up-regulation of several enzymes involved in starch degradation seems consistent with the need to speed up the use of energy reservoirs under stress [31]. Starch biosynthesis is tightly correlated with photosynthesis, another process strongly affected by the environment; a major effect of drought is to reduce transpiration through stomatal closure at the whole plant level. We identified 53 DEGs in total, including ten copies of BAM1, which were highly up-regulated with 3.4- to 8.9-fold change expressions in all genotypes expect Msin-G2, where we obtained much less DEGs. During osmotic stress, starch is degraded in the light by stress-activated BAM1 and AMY3 to release sugar and sugar-derived osmolytes [32, 33]. Abscisic acid controls the activity of BAM1 and AMY3 in leaves under osmotic stress through the AREB/ABF-SnRK2 kinase-signalling pathway [32]. We also observed a strong up-regulation of GBSS-I, which is involved in amylase synthesis [33], because a common trait of many plants affected by drought or salinity stress is the accumulation of osmoprotectants such as proline, glycine betaine, and sugar alcohols [34].

Twelve aquaporins were up-regulated across *Miscanthus* genotypes and associated with the enrichment of five GO terms associated with water and glycerol transport and homeostasis. Since many aquaporins (AQPs) act as water channels, they play an essential role in plant water and glycerol relations [35, 36]. *Miscanthus* aquaporins were homologous to multiple isoforms of PIP1, PIP2 and NIP2 in rice and *Arabidopsis.* The highest up-regulation was observed for PIP1-3 across genotypes, as well as in two uncharacterised aquaporins in specific genotypes. As observed, most plasma membrane intrinsic proteins (PIPs) have a higher level of expression than NOD26-like proteins (NIPs) in *Arabidopsis* [37]. The same paper observed variable regulation (up- or down-regulation) of specific aquaporins in drought stress [37]. However, we observed all of them up-regulated in *Miscanthus*. Another study [38] showed co-expression and physical interaction between PIP1 and PIP2 isoforms in heteromers.

Oxidation–reduction process was primarily up-regulated across genotypes, but some DEGs in this GO term were also down-regulated (Fig. 3). Up-regulation of genes involved in oxidation–reduction process was observed during drought in the wheat and sorghum transcriptomic analysis [39, 40].

Some of the GO terms were inconsistently enriched across *Miscanthus* genotypes. “RNA binding”, “translation”, “ribosome genesis” and “structural ribosome” were related and significantly enriched in the *M. sacchariflorus* (Msac-G1) and *M. x giganteus* (Mxg-G5) genotypes, but absent from the other two *Miscanthus* genotypes included in the study (Fig. 3). A previous study in *Arabidopsis* evidenced that different RNA binding proteins play a role in response to drought stress [41].

We observed the up-regulation of several GO terms related to biosynthetic pathways among all genotypes. Transcriptomics studies in diverse species during drought stress revealed up-regulation of biosynthetic pathways for phenolic acids and flavonoids, as well as the biosynthesis of multiple secondary metabolites that would act as antioxidants and minimise adverse effects of water deprivation [42–45].

## Conclusion

In the present study, a combination of phenotyping under greenhouse conditions and comparative gene expression analysis gave insight into the differential physiological and regulatory response to water stress, either flooded and droughted conditions, among genotypes from different *Miscanthus* species. The low number of DEGs in flooded conditions and a higher biomass yield observed in most genotypes in water-saturated conditions compared to the control, support that *Miscanthus* could be an option in marginal arable fields. For drought stress, different phenotypic responses were observed among *Miscanthus* species, suggesting differences in genetic adaption. This study is the first attempting to identify genes playing key roles in response to water stress across and between *M. sinensis, M. sacchariflorus*, and their natural and induced hybrids, *M. x giganteus* and a newly bred triploid *M. sinensis x M. sacchariflorus* hybrid genotype. We observed that the same biological processes were regulated across species during drought stress despite the DEGs were not necessarily the same ones. The critical role of starch metabolism (BAM1, AMY3, ISA3, GBSS-I, SUS3, SPS1F, SS3, BE1, SEX1), cellulose metabolism (CESA4, IRX1/3) and aquaporins (PIP2-1, PIP2-2, PIP2-7, PIP1-1, PIP1-3, ERECT1) noticed in *Miscanthus* species was consistent with functional categories known to be critical during drought stress in other organisms. *Miscanthus* also can offer a relevant model to study the differences in expression resulting from ploidy and heterosis.

## Material and methods

### Plant growth conditions

All genotypes were grown in three conditions (“control”, “drought” and “flooded”) in the glasshouse. The decision on treatments was taken empirically based on a small pre-experiment with *Miscanthus*. Each condition was repeated in each of four blocks placed in a glasshouse in a randomised block design. Each genotype was represented by two plants, each in a separate pot. One pot was used for biomass weight measurement (fresh and dry weights) at the end of the experiment, hence untouched. While the other pot was used for taking leaf samples for electrolyte leakage and relative water content (RWC) measurements. All measurements were conducted at the end of the experiment unless stated otherwise.

#### Plant materials

The physiological experiment was carried out on six *Miscanthus* genotypes which were clonally multiplied by Cora Münnich at Tinplant GmbH/Germany. Genotype Msac-G1: *M. sacchariflorus* DK-1 (received from Uffe Jørgensen/ Aarhus University) (tetraploid), genotype Msin-G2: *M. sinensis* accession 48 (Teagasc collection received from Trinity College Dublin as unnamed clone) (diploid), Genotype Msac-G3: *M. sacchariflorus* (Teagasc collection) (tetraploid), Genotype Mxg-G4: *M. x giganteus* (from Trevor Hodkinson/Trinity College, Dublin) (triploid), Genotype Mxg-G5: *M. x giganteus* ‘Illinois’ (received from Mike Jones/Trinity College Dublin)(triploid), and Genotype Hyb-G6: newly bred *M. sacchariflorus x M. sinensis* interspecific triploid hybrid S88 (bred by Svaloef Weibull/ Sweden).

### Water stress analysis

The plants were received from Tinplant GmbH as plantlets with ca 50 cm height on average 4 weeks before the experiment started. They were transplanted from 6cm × 6cm pots on 11/08/2013 in 10 × 10 cm pots and were left to grow to ~ 60 cm height prior to the start of the treatments. The temperature in the glasshouse during the day (09:00–17:00 h GMT) was set at 21 °C and during the night (17:00–09:00 h GMT) at 17 °C. The roof and walls of the glasshouse provided an abundance of natural light between August and September. Differential water conditions started on 19/08/2013. “Control” samples received 100 ml water, pots standing on capillary matting. Drought-treated plants received 40 ml water, standing on saucers to avoid uptake of water from capillary matting. Pots within flooded treatment were kept in trays with a continuous water level of between 5 and 8 cm water. The treatment effects on plants were measured via relative water content of leaves and electrolyte leakage of leaves. At the end of the experiments the plants were harvested above the soil and weighed for the determination of fresh and dried biomass (see “Biomass monitoring”).

### Soil moisture measurements

Soil moisture measurement was carried out in one of the pots representing each accession for each of the four blocks during the running of the experiment. A total of 17 measurements were recorded during the experiment. Soil moisture was measured with a Theta Kit soil moisture instrument from Delta-T Devices Ltd (Cambridge, UK). Three independent measurements were averaged to determine the soil moisture per pot. Values per pot were averaged for each genotype. The mean moisture content per condition is shown in Additional file 17: Figure S5.

### Relative water content of leaves (RWC) analysis

The highest leaf was collected and 5 cm from the leaf tip were cut and weighed for the fresh weight (FW). The leaf was submerged in 20 ml distilled water and left in the refrigerator for 24 h. The turgid weight (TW) was determined by blotting the leaf dry before weighing. The leaf was dried for 48 h at 80 °C and weighed again for the dry weight (DW). The RWC was calculated using the formula (FW-DW)/(TW-DW) × 100 = % RWC. Measurements were taken at two time points: 13/09/2013 and 24/09/2013, i.e. 4 weeks (33 days) and 6 weeks (44 days) after water treatment, respectively. The first time point was chosen once the soil water curve sloped visibly and the second time point was at the end of the experiment.

### Electrolyte leakage analysis

Two leaves were placed in a 50-ml polypropylene tube filled with distilled water. The tubes were closed, covered in tinfoil for darkness, and left for 24 h at room temperature. After 24 h conductivity was measured with a Hanna Instruments EC215 multi-range conductivity meter, after which the samples were autoclaved at 120 °C for 15 min. After cooling to room temperature, the conductivity of the solutions was measured again. The percentage of conductivity was calculated as ratio of conductivity before and after autoclaving, the latter representing 100% leakage. Measurements were taken on 25/09/2013 after 45 days in the experiment.

### Biomass monitoring

Fresh weight was determined for total plant above the soil. Samples for fresh and dry biomass were taken on 25/09/2013. Dry weight was determined after drying the fresh biomass for 48 h at 80 °C.

### Statistical analysis of the phenotyping

Factors in the phenotypic analyses included block, treatment and time. Logarithmic values were used for analysis of variance (ANOVA) between treatment groups. Where measurements of a response were made at a number of time points, these were included in the analysis as repeated measures and the correlations were modelled using a covariance structure in the Mixed procedure in SAS [46]. Where appropriate, baseline measurements were used as covariates. Tukey adjustments for multiplicity were used for means comparisons and residuals were checked to ensure that the assumptions of the analyses were met.

### RNA-sequencing

Four genotypes (*M. sacchariflorus* Msac-G1, *M. sinensis* Msin-G2, *M. x giganteus* Mxg-G5 and the interspecific triploid hybrid Hyb-G6) were selected towards the end of the experiment and sequenced in 2014. Leaf samples from the second leaf from top were taken on 12/09/2013, towards the end of the experiment, and flash-frozen in liquid nitrogen.

Total RNA was extracted using Qiagen’s RNeasy plant Mini kit according to the manufacturer’s instruction, including an on-column digestion of residual genomic DNA. The total RNA was converted into mRNA sequencing libraries using the Illumina TruSeq RNA Sample Preparation Kit (V2) according to the manufacturer’s instructions. Three biological replicates were taken for each genotype within each treatment group. Therefore, a total of 36 independent libraries were sequenced as 100 bp paired-end reads on an Illumina HiSeq 2000 sequencer. The libraries were multiplexed six times in one sequencing flow cell lane, using six lanes. All raw sequencing data were submitted to ArrayExpress (accession number E-MTAB-9354).

### RNA-seq reads, pre-processing and alignment

FastQC (v. 11.5) with default parameters was used to assess raw reads quality in each *Miscanthus* RNA-seq each library separately [47]. Thereafter, adapter sequences and low-quality reads were trimmed with Trimmomatic (v. 0.38) [48]. All subsequent analyses were performed on reads with a Phred score over 30 and minimal length of 36 bases. Clean reads were aligned to the *M. sinensis* reference genome (*M. sinensis* v7.1 DOE-JGI, http://phytozome.jgi.doe.gov) downloaded from Phytozome with STAR using the “2-pass” mode [49]. The reference was indexed using the *M. sinensis* gene annotation (*M. sinensis* v7.1 DOE-JGI, http://phytozome.jgi.doe.gov) downloaded from Phytozome in GFF3 format. This same gene annotation was functionally annotated with GO terms and enzyme codes with the command-line version of Blast2GO [50] using BLASTX with an E-value of 1e-10 and the NCBI non-redundant (nr) and EBI InterPro databases.

### Differential expression and enrichment in gene ontology (GO) terms analysis

The differential expression and enrichment analysis are fully available in an R notebook [51]. Counts were estimated with Stringtie for each genotype [52]. Differential expression analysis of each treatment against the control group was performed using the DESeq2 R package based on the negative binomial distribution model [53]. Genes with *P*-value < 0.05 adjusted by Benjamini and Hochberg’s method [54] were considered differentially expressed (DEGs). DEGs shared among four genotypes were visualised with an UpSet diagram using the UpSetR package (v. 1.4) [55]. In order to display the effect of treatment on different species and conditions (e.g. drought), a PCA analysis was carried out with “prcomp” from R and ggplot2 [56].

Enriched GO terms and other categories in each group of differentially expressed genes were identified in R using TOPGO [57] using a Fisher’s test (FDR < 0.05) and the “weight01” algorithm from TOPGO. Using the lists of DE genes and functional annotation as inputs, topGO compared the number of DEGs in each category with the expected number of genes for the whole transcriptome. The “weight01” algorithm resolves the relations between related GO ontology terms at different levels. The relation among GO terms was plotted in R using ggplot [56]. Genes in enriched GO terms were further analysed in the online Phytomine [58] and Thalemine [59] databases. Genes annotated with enzyme codes were plotted using the online KEGG mapper [60].

## Supplementary Information

**Additional file 1: Figure S1.** Fresh and biomass weights for the six genotypes in non-transformed units.

**Additional file 2: Table S1.** Tukey–Kramer groupings for genotype × treatment Least Squares Means for the traits fresh weight biomass, dry weight biomass, electrolyte leakage and relative water content (Alpha = 0.05). LS-means with the same letter are not significantly different.

**Additional file 3: Table S2.** Basic information on sequencing, processing and mapping of the RNA-Seq libraries.

**Additional file 4: Table S3.** Gene read counts per library.

**Additional file 5: Table S4.** DEseq2-normalised gene read-counts per library.

**Additional file 6: Table S5.** Differential expression analysis between drought and control conditions.

**Additional file 7: Figure S2.** Number of differentially expressed genes (DEGs) shared within and among four *Miscanthus* species under flooded conditions.

**Additional file 8: Table S6.** Differential expression analysis between flooded and control conditions.

**Additional file 9: Table S7.** Functional annotation of the *M. sinensis* genome with Gene Ontology (GO) terms.

**Additional file 10: Table S8.** Enrichment analysis *full* GO: over-represented GO annotations among genes differentially expressed during drought.

**Additional file 11: Table S9.** Enrichment analysis GO-SLIM: over-represented GO-SLIM annotations among genes differentially expressed during drought.

**Additional file 12: Table S10.** Function, KEGG, and *Arabidopsis thaliana* homologous annotation for differentially expressed genes in significantly enriched GO categories.

**Additional file 13: Figure S3.** GO SLIM terms (rows) that were significantly enriched (p < 0.005) in each *Miscanthus* species (columns) among either up-regulated (top-pointing triangles) or down-regulated (bottom-pointing triangles) differentially expressed genes (DEGs) in drought conditions. The size of a triangle is proportional to the number of DEGs annotated with that GO term. Rows are sorted by descending p-value (F-Fisher test) and the triangle colour is representative to the obtained p-value, from lower (dark colour) to higher (light colour). Yellow (p > 0.05) and white (p > 0.1) triangles were not significantly enriched.

**Additional file 14: Table S11.** Candidate genes in the starch and sucrose pathways highlighted by our analysis.

**Additional file 15: Figure S4.** Reactions in the starch and sucrose metabolic pathways that were up-regulated (red boxes) during drought stress in at least one of the analysed *Miscanthus* genotypes.

**Additional file 16: Table S12.** Candidate genes in the water/glycerol transport highlighted by our analysis.

**Additional file 17: Figure S5.** Mean soil moisture readings for all genotypes per condition across 17 days.

## Data Availability

The RNA-seq data have been assigned ArrayExpress accession E-MTAB-9354. The R code used in the analysis is deposited in Zenodo (http://doi.org/10.5281/zenodo.3950495) and Github (https://joseja.github.io/miscanthus_drought_rnaseq/).
