# Supplementary figures and images for: Physiological and transcriptional response to drought stress among bioenergy grass Miscanthus species
Source: Biotechnol Biofuels. 2021 Mar 6;14:60. doi: 10.1186/s13068-021-01915-z (PMC7937229; doi:10.1186/s13068-021-01915-z)

*Msac-G1*   *Msac-G3*   *Mxg-G5*  
*Msin-G2*   *Mxg-G4*   *Hyb-G6*

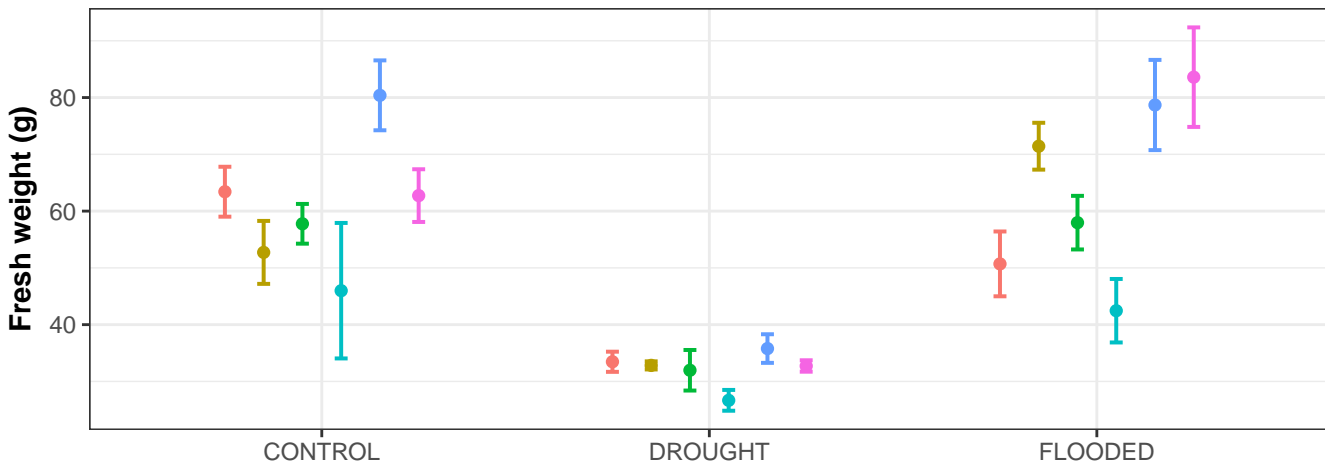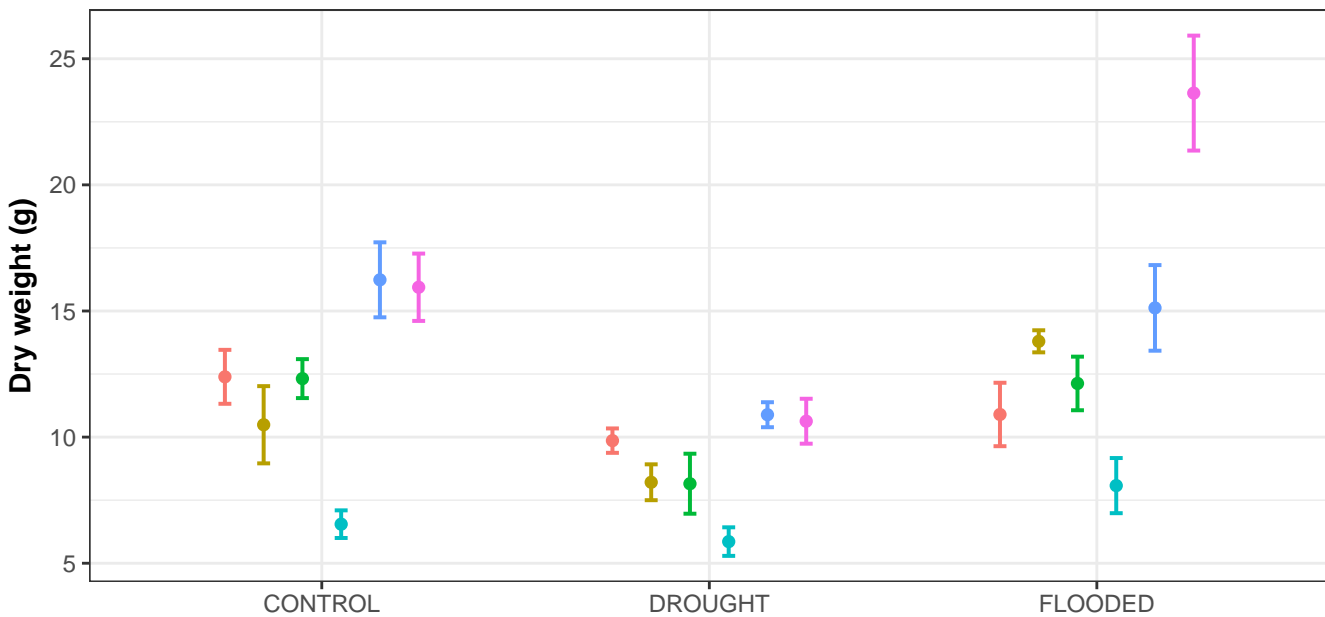

Supplement: Supplementary file 1 — Additional file 1: Figure S1. Fresh and biomass weights for the six genotypes in non-transformed units. [file 13068_2021_1915_MOESM1_ESM.pdf]

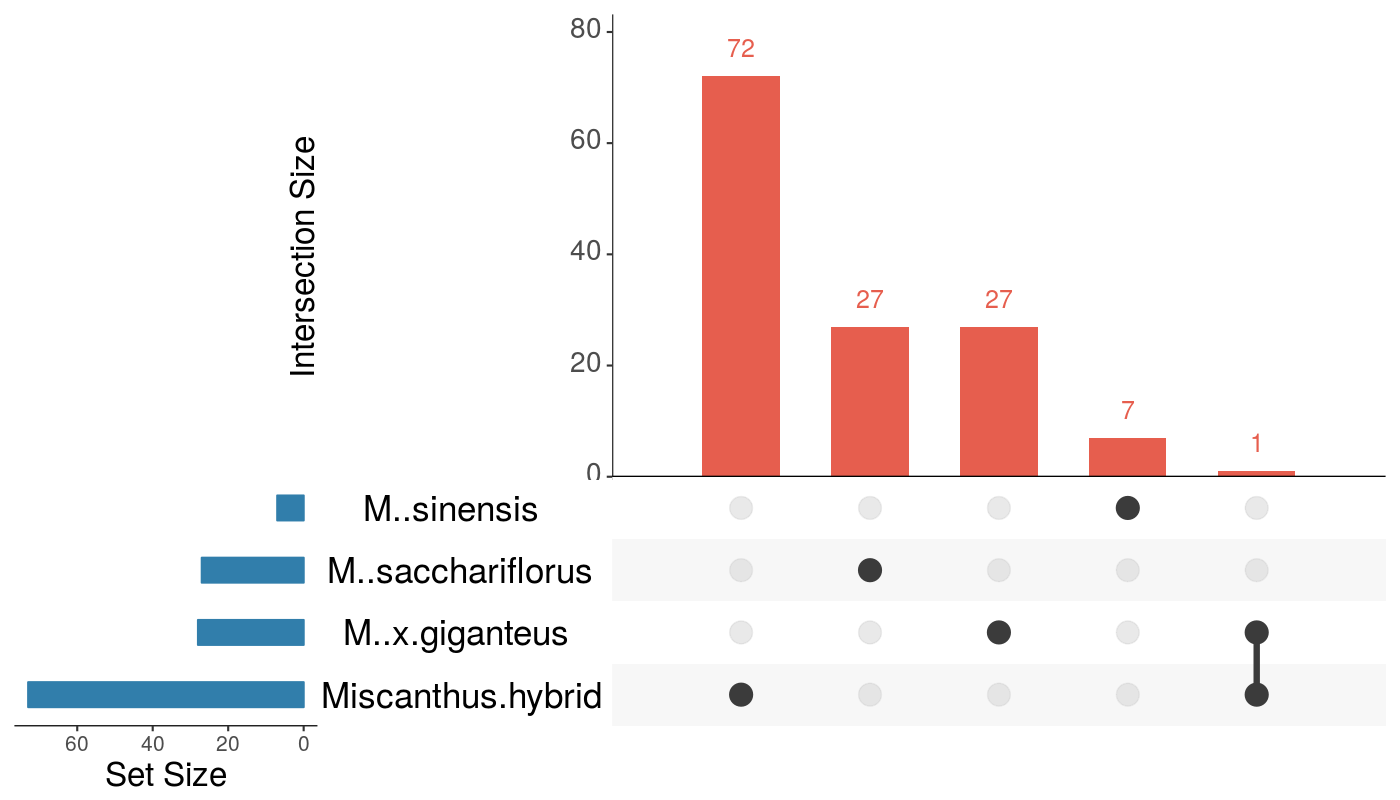

Supplement: Supplementary file 7 — Additional file 7: Figure S2. Number of differentially expressed genes (DEGs) shared within and among four Miscanthus species under flooded conditions. [file 13068_2021_1915_MOESM7_ESM.png]

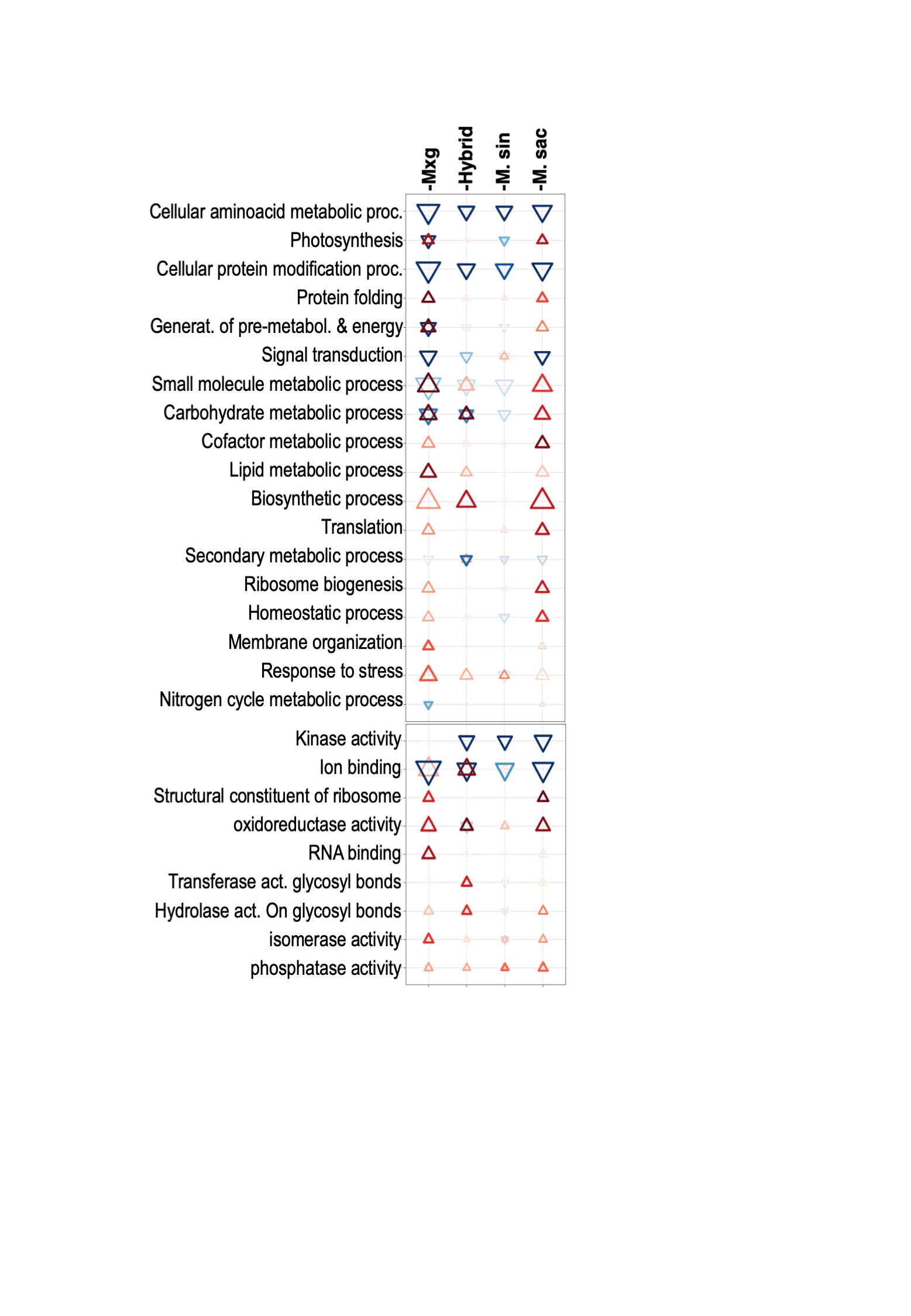

Supplement: Supplementary file 13 — Additional file 13: Figure S3. GO SLIM terms (rows) that were significantly enriched (p < 0.005) in each Miscanthus species (columns) among either up-regulated (top-pointing triangles) or down-regulated (bottom-pointing triangles) differentially expressed genes (DEGs) in drought conditions. The size of a triangle is proportional to the number of DEGs annotated with that GO term. Rows are sorted by descending p-value (F-Fisher test) and the triangle colour is representative to the obtained p-value, from lower (dark colour) to higher (light colour). Yellow (p > 0.05) and white (p > 0.1) triangles were not significantly enriched. [file 13068_2021_1915_MOESM13_ESM.png]

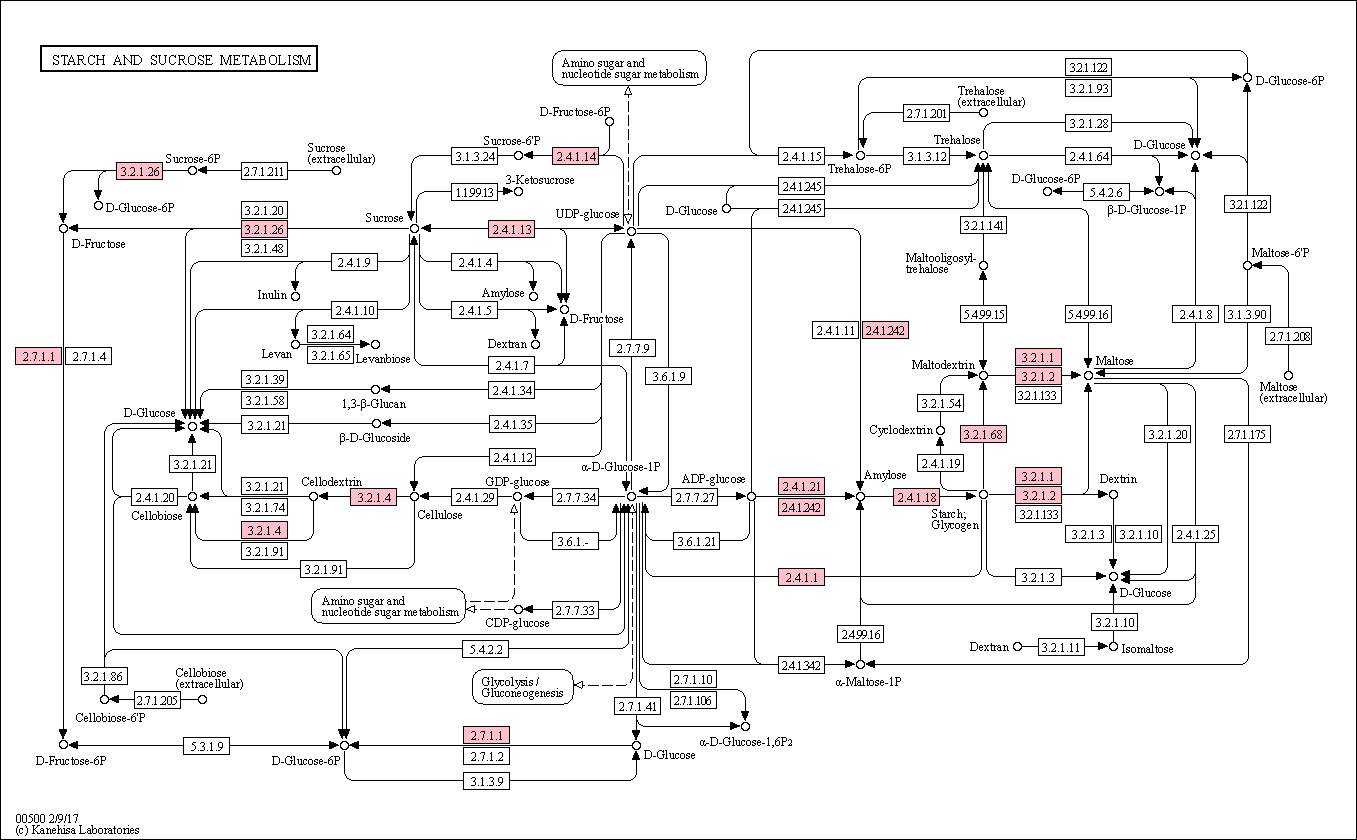

Supplement: Supplementary file 15 — Additional file 15: Figure S4. Reactions in the starch and sucrose metabolic pathways that were up-regulated (red boxes) during drought stress in at least one of the analysed Miscanthus genotypes. [file 13068_2021_1915_MOESM15_ESM.png]

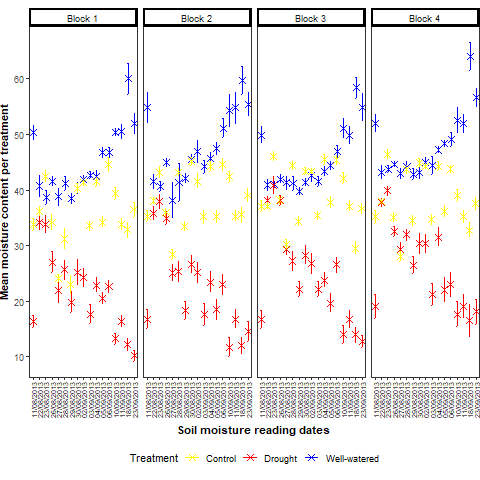

Supplement: Supplementary file 17 — Additional file 17: Figure S5. Mean soil moisture readings for all genotypes per condition across 17 days. [file 13068_2021_1915_MOESM17_ESM.png]
