# Supplementary material for: Physiological and transcriptional response to drought stress among bioenergy grass Miscanthus species
Source: Biotechnol Biofuels. 2021 Mar 6;14:60. doi: 10.1186/s13068-021-01915-z (PMC7937229; doi:10.1186/s13068-021-01915-z)
Supplement: Supplementary file 2 — Additional file 2: Table S1. Tukey–Kramer groupings for genotype × treatment Least Squares Means for the traits fresh weight biomass, dry weight biomass, electrolyte leakage and relative water content (Alpha = 0.05). LS-means with the same letter are not significantly different. [file 13068_2021_1915_MOESM2_ESM.docx]

Tables S1: Tukey-Kramer groupings for genotype x treatment Least Squares Means for the traits fresh weight biomass, dry weight biomass, electrolyte leakage and relative water content (Alpha = 0.05). LS-means with the same letter are not significantly different.

Fresh weight biomass

| Genotype | Treatment | Estimate | Grouping |
| --- | --- | --- | --- |
| G2 | CONTROL | 2.03 | A |
| G1 | CONTROL | 1.98 | AB |
| G3 | CONTROL | 1.98 | AB |
| G4 | CONTROL | 1.97 | AB |
| G5 | CONTROL | 1.93 | AB |
| G6 | CONTROL | 1.93 | AB |
| G4 | DROUGHT | 2.04 | A |
| G2 | DROUGHT | 2.01 | AB |
| G1 | DROUGHT | 1.99 | AB |
| G6 | DROUGHT | 1.97 | AB |
| G3 | DROUGHT | 1.76 | BC |
| G5 | DROUGHT | 1.66 | C |
| G5 | FLOODED | 2.16 | A |
| G6 | FLOODED | 2.11 | A |
| G1 | FLOODED | 2.10 | A |
| G2 | FLOODED | 2.06 | A |
| G3 | FLOODED | 1.98 | AB |
| G4 | FLOODED | 1.97 | AB |

Dry weight biomass

| Genotype | Estimate | Grouping |
| --- | --- | --- |
| G6 | 2.82 | A |
| G5 | 2.68 | AB |
| G1 | 2.47 | BC |
| G3 | 2.44 | C |
| G2 | 2.43 | C |
| G4 | 2.03 | D |

Electrolyte leakage

| Genotype | Treatment | Estimate | Grouping |
| --- | --- | --- | --- |
| G2 | CONTROL | 2.03 | A |
| G1 | CONTROL | 1.98 | AB |
| G3 | CONTROL | 1.98 | AB |
| G4 | CONTROL | 1.97 | AB |
| G5 | CONTROL | 1.93 | AB |
| G6 | CONTROL | 1.93 | AB |
| G4 | DROUGHT | 2.04 | A |
| G2 | DROUGHT | 2.01 | AB |
| G1 | DROUGHT | 1.99 | AB |
| G6 | DROUGHT | 1.97 | AB |
| G3 | DROUGHT | 1.76 | BC |
| G5 | DROUGHT | 1.66 | C |
| G5 | FLOODED | 2.16 | A |
| G6 | FLOODED | 2.11 | A |
| G1 | FLOODED | 2.10 | A |
| G2 | FLOODED | 2.06 | A |
| G3 | FLOODED | 1.98 | AB |
| G4 | FLOODED | 1.97 | AB |

Relative water content

| Genotype | Treatment | Estimate | Grouping |
| --- | --- | --- | --- |
| G2 | CONTROL | 2.03 | A |
| G1 | CONTROL | 1.98 | AB |
| G3 | CONTROL | 1.98 | AB |
| G4 | CONTROL | 1.97 | AB |
| G5 | CONTROL | 1.93 | AB |
| G6 | CONTROL | 1.93 | AB |
| G4 | DROUGHT | 2.04 | A |
| G2 | DROUGHT | 2.01 | AB |
| G1 | DROUGHT | 1.99 | AB |
| G6 | DROUGHT | 1.97 | AB |
| G3 | DROUGHT | 1.76 | BC |
| G5 | DROUGHT | 1.66 | C |
| G5 | FLOODED | 2.16 | A |
| G6 | FLOODED | 2.11 | A |
| G1 | FLOODED | 2.10 | A |
| G2 | FLOODED | 2.06 | A |
| G3 | FLOODED | 1.98 | AB |
| G4 | FLOODED | 1.97 | AB |
